# New strategies for producing defect free SiGe strained nanolayers

**DOI:** 10.1038/s41598-018-21299-9

**Published:** 2018-02-13

**Authors:** Thomas David, Jean-Noël Aqua, Kailang Liu, Luc Favre, Antoine Ronda, Marco Abbarchi, Jean-Benoit Claude, Isabelle Berbezier

**Affiliations:** 1 CNRS, Aix Marseille University, UMR 7334, Inst Mat Microelect Nanosci Prov, F-13397 Marseille, France; 2 University Paris 06, CNRS UMR 7588, Inst Nanosci Paris, F-75252 Paris, France

## Abstract

Strain engineering is seen as a cost-effective way to improve the properties of electronic devices. However, this technique is limited by the development of the Asarro Tiller Grinfeld growth instability and nucleation of dislocations. Two strain engineering processes have been developed, fabrication of stretchable nanomembranes by deposition of SiGe on a sacrificial compliant substrate and use of lateral stressors to strain SiGe on Silicon On Insulator. Here, we investigate the influence of substrate softness and pre-strain on growth instability and nucleation of dislocations. We show that while a soft pseudo-substrate could significantly enhance the growth rate of the instability in specific conditions, no effet is seen for SiGe heteroepitaxy, because of the normalized thickness of the layers. Such results were obtained for substrates up to 10 times softer than bulk silicon. The theoretical predictions are supported by experimental results obtained first on moderately soft Silicon On Insulator and second on highly soft porous silicon. On the contrary, the use of a tensily pre-strained substrate is far more efficient to inhibit both the development of the instability and the nucleation of misfit dislocations. Such inhibitions are nicely observed during the heteroepitaxy of SiGe on pre-strained porous silicon.

## Introduction

During the past decades the field of IV-IV SiGe/Si heterostructures has witnessed dramatic advances thanks to extensive research along new pathways to enhance their electronic properties, in particular using strain engineering nanostructures to largely confine the carriers^1–6^. The increasing role of group IV nanostructrues in quantum electronics and photonic devices is due to their excellent compatibility with CMOS technology, ease of integration and perfect strain control^7–10^. The epitaxial strain induced by the mismatch between SiGe and Si is still considered as the most efficient way to produce strain engineering that is a promising path for drive current enhancement by improving the electron and hole mobility in Si-based devices^11,12^. Furthermore, most of the physical and structural properties of SiGe can be continuously tuned with composition from 0 to 100% Ge concentration. This is particularly true for the lattice constant and epitaxial strain which varies linearly with Ge concentration offering a cost-efficient way to produce controlled strain-engineering systems in order to controllably enhance electronic transport properties like charge carrier mobility, carrier confinement effects etc^13,14^.

Various systems including superlattices, core-shell nanowires, quantum dots, nanocrystals that have been suggested as high-performance materials have failed to meet that challenges mainly because of the strain-related behaviours such as elastic relaxation by Asarro Tiller Grinfled (ATG) growth instability or Ge hut islands nucleation, plastic relaxation by nucleation of dislocations and chemical relaxation by strain-driven interdiffusion^15^.

The use of a compliant substrate for the epitaxy of SiGe layers could be an elegant and low cost solution to control the strain-related behaviour^16–18^. In this context, the role of a template layer on the strain distribution and as a consequence on the ATG development and the nucleation of dislocations should be fully understood both theoretically and experimentally. The template layer with specific elastic properties could be either soft/rigid and/or pre-strained, acting as a compliant substrate.

Alternative approaches have been explored to enhance devices performances. In particular, nMOS and pMOS devices are now built on Silicon On Insulator (SOI) substrates. Their improved performances result mainly from the reduction of parasitic device capacitance, and leakage current. In addition, the introduction of strained silicon, in particular with the epitaxial growth of Si on Silicon Germanium (SiGe) fully strained layers, could significantly improve the mobility of both n and p channel devices. Moreover, fabrication of ultra-thin Germanium On Insulator (GOI) substrates plays also an important role in <10 nm technology node and beyond. In such approaches, it is mandatory to determine the level of biaxial strain in Si and SiGe layers and to predict the strain relaxation (Ge interdiffusion, dislocation nucleation etc.) induced by subsequent processing steps. In parallel, it has been suggested that SOI could behave as a compliant substrate which could suppress the development of the ATG instability and allow full strain relaxation by fluency gliding at the interface^19–24^. Epitaxial SiGelayers on buried oxide are then under intense scrutiny due to their applications in ultra-scales performance-augmented CMOS transistors^25–29^. The aim is to pursue the Moore’s law by developing planar transistors based on ultra-thin Si/SiGe or pure Ge nanochannels on insulator (UTSGOI or UTGOI) systems^30–34^. One major hurdle is the elaboration of Ge rich layers fully planar and strained with perfectly controlled homogeneous strain, thickness and composition^35,36^.

So, the stability of SiGe layers and the mechanisms of strain relaxation in Silicium Germanium On Insulator (SGOI) are issues of crucial importance for the fabrication of the next generation fully depleted CMOS devices^25,33,37–39^. Further understanding of the strain relaxation on a soft buried oxide is then desired.

In order to experiment over a wider range of parameters, porous silicon was used to provide an archetype model system since it has tunable elastic properties that controllably depend on experimental parameters (mainly pores density and shape)^40–43^. Moreover, thanks to its unique properties such as distinctive photoluminescence, biocompatibility and active surface properties porous silicon could be compatible with Si microelectronic technology^44^.

Our work mainly addresses the evolution of strain relaxation in SGOI layers. The effect of softness and pre-strain of the substrate on strain relaxation was studied. A combined theoretical/experimental study was carried out to determine the morphological evolution and nucleation of dislocation of epitaxial layers on soft SOI and porous silicon substrates. With a combination of well-chosen experimental and theoretical demonstrations we show that a compliant substrate (CS) could considerably modify the growth modes of heteroepitaxial layers. We first show theoretically that an elastically soft (rigid) substrate free of strain would enhance (reduce) the development of the growth instability while not affecting the nucleation of dislocations. In the case of SiGe heteroepitaxy, because of the presence of a thin silicon template layer (necessary for the epitaxial growth), the softness of the compliant substrate has almost no effect on the ATG instability. These theoretical predictions match experimental results on SOI and on porous silicon substrates.

With this work we normalize most of the experimental findings on the effect of compliant substrates reported in the literature. The combination of theoretical and experimental results provides new thinking dealing with substantive issues and methodologies that will direct and orient the process development for the implementation of SGOI systems into CMOS application. They can lead to the evolution of a new paradigm valid for other high-end systems.

## Experimental

SiGe thin films were deposited by molecular beam epitaxy (MBE) on top of two different types of substrates made either of Silicon On Insulator (SOI) or Porous Silicon (PSi).

The SOI substrates (CEA-Leti, France) were fabricated by the Smart Cut(TM) process. They are single-crystal Si(001) films, with thickness 10 nm, bonded to a 12 nm thick SiO_2_ layer on a standard Si(001) wafer. The porous silicon substrates were obtained by electrochemically etching of B-doped <100>-oriented Si wafers in a hydrofluoric acid (HF) solution^45^. The porous silicon layers used in this study have been fabricated by STMicroelectronics. They have a mean porosity ~60% (which corresponds to the maximum density of pores on which good reepitaxy can be processed). Experimental details of the electrochemical process are given elsewhere^46,47^.

Immediately after the electrochemical formation step, either the samples are loaded in the MBE machine after *ex situ* chemical cleaning (the samples are then named: PSi) or the samples are heated *ex situ* at high temperature between 900 °C and 1100 °C (they are then named: HTPSi), then chemically cleaned and loaded in the MBE machine after *ex situ* chemical cleaning. We used these two kind of porous silicon substrates, as grown (PSi) and after high temperature annealing (HTPSi). While PSi and HTPSi substrates have the same softness, the samples treated at high temperature (HTPSi) are tensily pre-strained.

The *ex situ* cleaning process follows a modified Shiraki recipe: (i) 10 min in HNO3 (65%) heated at 70 °C, (ii) 1 min in deionized water, and (iii) 30 s in HF (49%): H2O (1:10). To avoid contamination, the substrates are immediately introduced into the UHV MBE growth chamber RIBER MBE32 at the end of the chemical cleaning. Subsequently, the samples are thermally cleaned *in situ* at temperatures ~400 °C for 15 min before growth. They are then capped by a thin Si buffer layer 20 nm thick, which guarantees a flat and reproducible top surface. The mean root square roughness obtained after the buffer layer growth is similar to those of Si(0 0 1). This is followed by the epitaxial growth of SiGe layers of different thickness using solid source molecular beam epitaxy with a background pressure in the 10^−11^ torr range. Si was evaporated from an electron gun evaporator and Ge is evaporated from an effusion cell. Beam flux and SiGe compositions are calibrated *in situ* by reflection high energy electron diffraction (RHEED) oscillations. The Si buffer layer is deposited at 700 °C, while SiGe layers are deposited at 550 °C. At the end of the fabrication process, all the samples are containing the CS i.e.SOI or porous silicon, the Si buffer layer and the SiGe thin film.

After fabrication, all the samples were observed by atomic force microscopy (AFM), using a PSIA XE-100. AFM was used in noncontact operation mode in air, with a NCHR-50 tip model for very high-resolution imaging; its typical radius is about 8 nm.

Details on the structures are obtained by Transmission Electron Microscopy (TEM) using a FEI Tecnai G2 and a FEI Titan 80–300 with Cs corrector in TEM and Scanning Transmission Electron Microscopy (STEM) modes. Cross-section samples are prepared using a dual-beam FIB HELIOS 600 nanolab or tripod polishing followed by PIPS thinning.

## Results and Discussion

We first examine a geometry similar to the experimental configuration where the effect of the stiffness of the compliant substrate is maximal. We thence consider a three-layer system with a semi-infinite compliant substrate (CS), a thin silicon buffer layer (of thickness e) and an epitaxial SiGe layer (of thickness h). Details and ingredients of the theoretical model were given elsewhere^48^. The SiGe film has a lattice parameter (*a*^*SiGe*^) different from Si(*a*^*Si*^), with the misfit $m=\frac{({a}^{SiGe}-{a}^{Si})}{{a}^{Si}}$. For simplification, we consider that the CS has the same lattice parameter as Si. The system is supposed to be coherent and to be described by linear isotropic elasticity in the different layers and we only account for the difference in Young’s modulus^49–51^. While the SiGe and Si Young’s modulus are supposed to be similar (equal to Y), the CS stiffness is *Y*^*CS*^ = *sY*, with 0.1 ≤ s ≤ for a relatively soft CS and 1 ≤ *s* ≤ 10 for a rigid substrate. The displacement vector is solution of the equilibrium equation ∇.*σ* = 0 where *σ* is the stress tensor, with the boundary condition of a stress-free film surface *σ*.*n* = 0 where *n* is the normal to the surface.

In the case of a flat film (with a free surface defined by $z=\bar{h}=e+h$), the forces, the stress tensor, the displacement gradients, etc., are independent of *x*and *y* and the general solution for the Navier equation is merely:

1 $${u}_{0}=a.R+b$$

with a constant tensor *a* and vector *b*. It is associated with an energy density in the film:

2 $$\overline{{\varepsilon }_{0}}=Y{m}^{2}/(1-\nu )$$

When the film surface is corrugated, it is convenient to search for *u* in Fourier space along *r* = (*x*, *y*). We thence consider a free surface defined by $=\,\bar{h}+{h}_{1}{e}^{ikr}$, with the wavevector *k*. The solution of the Navier equation is: *u* = *u*_0_ + *u*_1_ where ${u}_{1}={h}_{1}{e}^{ikr}{\hat{u}}_{1}$ may be found exactly in the small-slope approximation. We eventually find that the elastic energy density on the surface is at first order:

3 $$\varepsilon ={\bar{\varepsilon }}_{0}-2(1+\nu ){\bar{\varepsilon }}_{0}{\rm{{\rm A}}}(k\bar{h})k{h}_{1},$$

where:

4 $$\begin{array}{rcl}A(x) & = & \frac{1}{\gamma (x)}\{[3-4\nu +(3-4\nu ){s}^{2}+2(8{\nu }^{2}-12\nu +\,5)s]{e}^{4x}\\  &  & +\,4(s-1)(3-4\nu +s)x{e}^{2x}+(4\nu -3){(s-1)}^{2}\}\end{array}$$

with:

5 $$\begin{array}{rcl}\gamma (x) & = & [(3-4\nu )(1-s)-(3-4\nu +s){e}^{2x}][1-s+(4\nu s-3s-1){e}^{2x}]\\  &  & +\,4{x}^{2}(s-1)(3-4\nu +s){e}^{2x}\end{array}$$

The elastic energy density depends both on the Young’s modulus ratio *s *and on the total film/buffer thickness $\bar{h}$.

The model describes the driving force for the morphological instability which is the strain relaxation in the film. However, the energy gain due to the elastic relaxation in the film *δE*^*film*^ < 0 resulting from the film corrugation, is counterbalanced by an energetic cost in the substrate *δE*^*sub*^ > 0, which is lower than |*δE*^*film*^| leading to a favorable balance. Since the energetic cost in the substrate *δE*^*sub*^ is proportional to its Young’s modulus the development of the instability is favored on a soft substrate, leading to the instability enhancement while the opposite behavior occurs on a rigid substrate i.e. a lower development of the instability and higher strain energy in the system.

Moreover, the evolution by surface diffusion of the film surface *h*(*x*, *y*, *t*) is dictated by the general diffusion equation:

6 $$\frac{\partial \mu }{\partial t}=D{\rm{\Delta }}\mu $$

with the diffusion coefficient *D* and the chemical potential *μ* on the surface^52^. The latter is the sum of the elastic energy density on the surface and the capillarity energy cost −*γ*Δ*h* at first order, with the surface energy *γ*. With this set of equations, the harmonic initial condition:

7 $$z=\bar{h}+{h}_{1}{e}^{ikr}\,{\rm{evolves}}\,{\rm{as}}\,{h}_{1}={h}_{1}(0){e}^{\sigma t}$$

where the growth rate is:

8 $$\sigma (k;\bar{h},s,\eta /m)=A(k\bar{h}){(1-\eta /m)}^{2}{k}^{3}-{k}^{4}$$

where k is the wave-vector. If the *k*^4^-term is only affected by corrections due to the stiffness, the *k*^3^-term which drives the instability is multiplied by (i) 1/*s* related to the CS softness and (ii) (1−*η*/*m*)^2^ related to the CS pre-strain. The first effect clearly enhances the instability when *s* <1, while the second slows down the instability when a tensile pre-strain (*η* > 0) is present.

**Figure 1:**
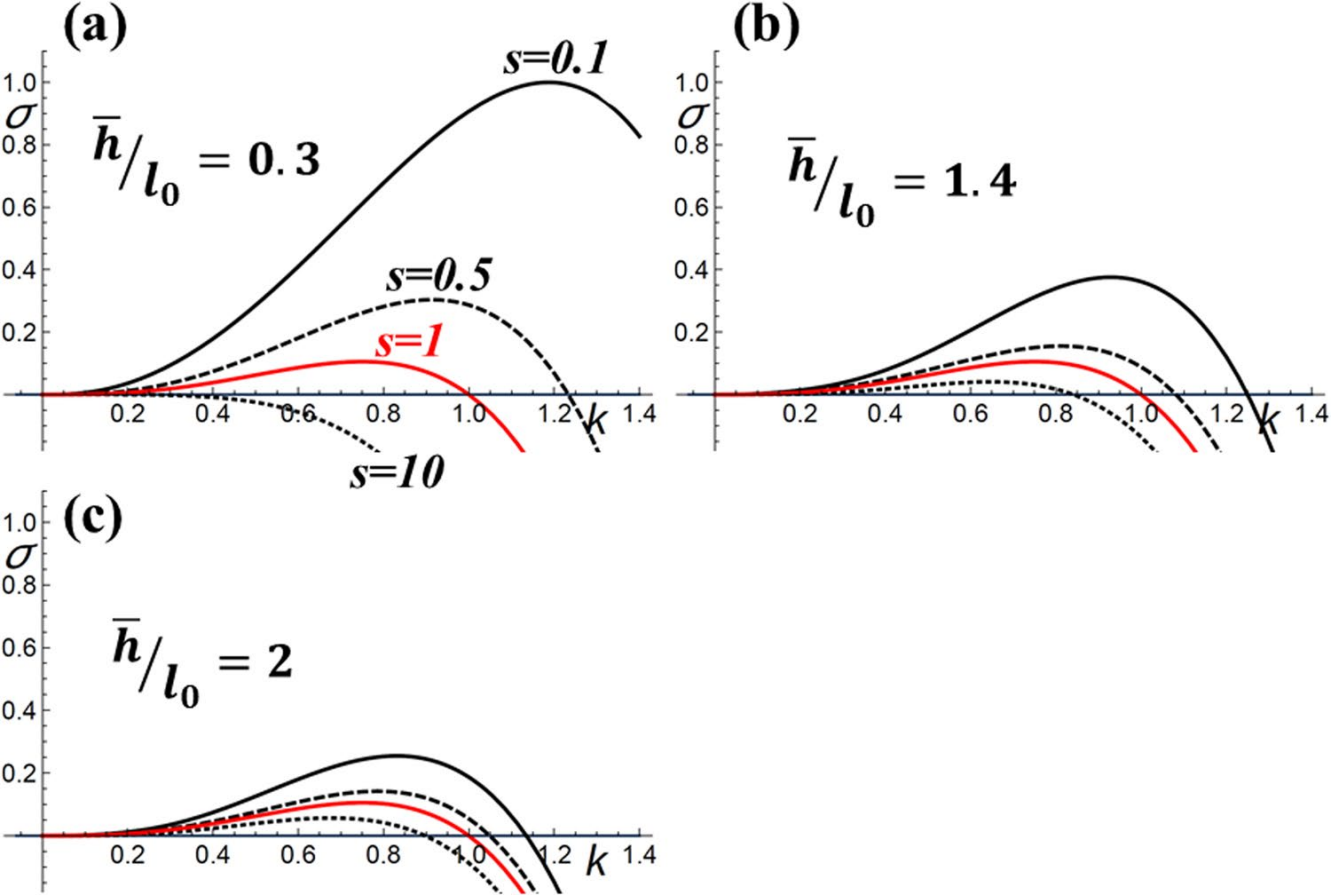
Evolution of the ATG growth rate (σ) for (**a**) $\bar{h}/{l}_{0}=0.3$; (**b**) $\bar{h}/{l}_{0}=1.4$; (**c**) $\bar{h}/{l}_{0}=2$ where the black solid line denotes for *s* = 0.1 (soft substrate); the black dashed line denotes for *s* = 0.5 (moderate soft substrate); the red solid line for *s* = 1 (bulk Si(001)); the black dotted line for *s* = 10 (rigid substrate).

If we consider the development of the instability for four values of s between *s* = 0.1 and *s* = 10, we see that the instability is considerably enhanced when *s* is small, i.e. for a softer CS (smaller Young’s modulus) and it is inhibited when *s* increases i.e. for a more rigid substrate (larger Young’s modulus). However, the amplitude of this effect is function of the total thickness $\bar{h}$ of the system (typically in our system it is the thickness of Si buffer + SiGe layer) rationalized by the wave-length of the instability $(\,\bar{h}/{l}_{0})$ (Fig. 1).

As a consequence, in standard conditions of SiGe heteroepitaxy (with a Si buffer layer *h* ~ 50 *nm* deposited prior to the epitaxy of the SiGe layer), when considering the critical thickness of ATG development (*h*_*ATG*_) for the whole range of Ge compositions, we find $1 < {\overline{h}}_{ATG}/{l}_{0}\le \,2\,$. In these experimental conditions, *s* is expected to have a much smaller effect on the development of the ATG instability (Fig. 1b–c) than for other systems that provide $\bar{h}/{l}_{0} < 1$. We can see for instance a strong enhancement (inhibition) of the ATG instability when *s* = 0.1 (*s* = 10) for $\bar{h}/{l}_{0}=0.3\,$ (Fig. 1a). We will see in the next parts that this condition is hard to reach with SiGe epitaxial layers. To evaluate this effect we consider experimentally two different substrates: SOI and porous silicon.

### Effect of a soft substrate: the case of silicon on insulator (SOI)

In order to determine the effect of the softness of the SOI substrate which is elastically softer than bulk Si we consider the epitaxy of SiGe on SOI. The elastic softness of the SiO_2_ underlayer gives (*s* ~ 0.5) and the system is initially unstrained (*η* = 0).

**Figure 2:**
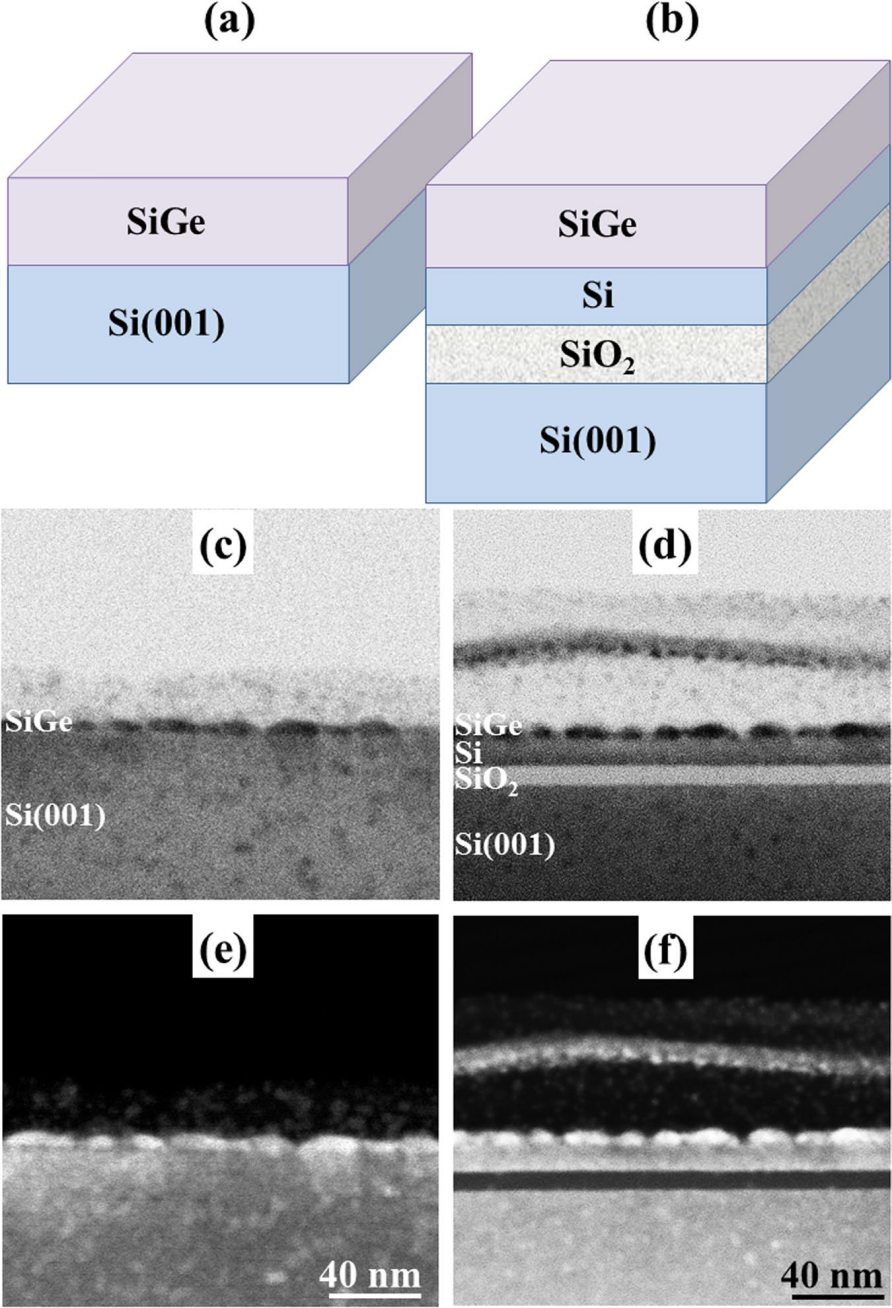
Schematic representation of the two systems: (**a**) Si_1−x_Ge_x_/Si and (**b**) Si_1−x_Ge_x_/SOI; Cross-section TEM (**c**) and (**d**) and ADF (**e**) and (**f**) images of Si_1−x_Ge_x_ epitaxial layers with h_SiGe_ = 10 nm and x = 0.5 when they are deposited on: Si(001) (left column) and SOI with Si top layer h_Si_ = 10 nm (right column). The height of the islands is about 5 nm.

In these conditions, we compare the growth of SiGe layers on SOI and on bulk Si(001). A schematic representation of the systems considered is given Fig. 2a,b. The aim is to determine the influence of the SOI softness on the development of the ATG instability. Experiments are carried out on different configurations of SOI substrates with SiO_2_ layer thickness varying from 10 nm to 240 nm. No effect of the SiO_2_ thickness could be observed. To facilitate the TEM visualisation of all the system’s layers, we only present here the results on ultra-thin SiO_2_ layer. We choose high concentration layers (*x* = 0.5) to exemplify the mechanism. The nominal deposited thickness of SiGe layers is chosen larger than the critical thickness for the development of the ATG instability (*h*_*Si*_ = 2 *nm*) but not too large to allow the softness of the substrate to provide an effect (*h*_*SiGe*_ = 10 *nm*). With an ultra-small Si buffer layer thickness (*h*_*Si*_ = 10 *nm*), it follows $\bar{h}=20\,nm$ and $\bar{h}/{l}_{0}=2$. The morphology of the SiGe surfaces is shown in Fig. 2.

While TEM conventional images are presented on Fig. 2c–f show Annular Dark Field (ADF) STEM images. This imaging mode gives a high chemical contrast which enlightens the SiGe layers morphology (bright layers on the images). We can clearly see that in the two situations the layers exhibit SiGe islands as commonly observed on Si(001) in these conditions. The corresponding bright field TEM images are given Fig. 2a and c.

**Figure 3:**
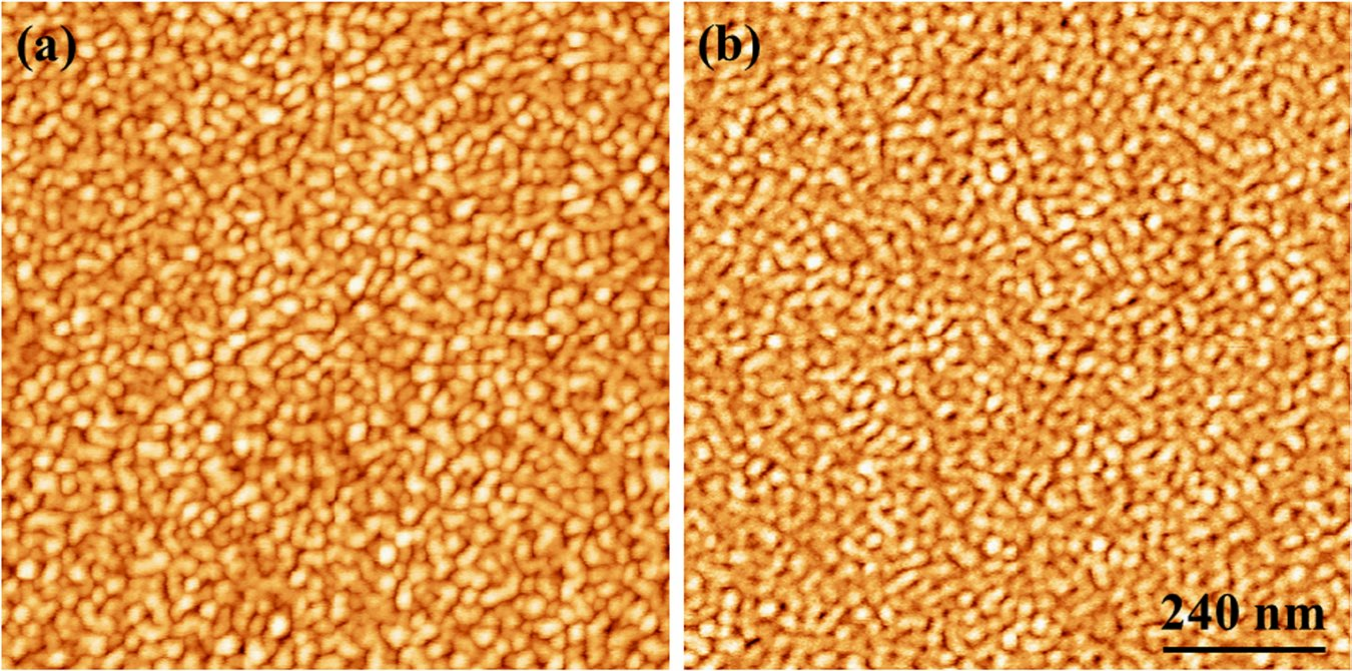
AFM topography images of SiGe layers with h_SiGe_ = 10 nm and x = 0.5 deposited on: (**a**) SOI and (**b**) Si(001). Scan size is 1 µm × 1 µm and height scale is 5 nm.

On a larger scale, the analysis of the surface morphology measured by AFM (Fig. 3) shows that the lateral size, the height and the density of the islands are almost the same in the two situations (~30 nm, ~3 nm and $1,5\cdot {10}^{11}/c{m}^{2}$ respectively). This demonstrates that the morphological evolution of the layers (resulting from the development of the ATG instability) is the same on the two substrates. Consequently in these conditions, the softness of SiO_2_ template layer cannot suppress the ATG instability. In these conditions, strain relaxation in the soft substrate is negligible as compared to bulk Silicon.

This experimental result is in good agreement with the theoretical results. The evolutions predicted by the simulations in the condition $\bar{h}/{l}_{0}=2$ (Fig. 1c), for *s* ~ 1 and *s* ~ 0.5, comparison of the black-dashed curve and red curve, are almost similar. The effect of the non-slippery (totally rigid) Si/SiO_2_ interface, which is not considered here, does not modify significantly the morphological evolution of the SiGe layer even if further simulations and experimental results would be needed to describe this situation.

### Effect of a soft substrate: the case of porous silicon

In order to check on a larger range the effect of the softness, in this part we used porous silicon (PSi) whose Young modulus can be largely varied with the density of pores. In particular, a Young modulus two orders of magnitude smaller than bulk silicon (s = 0.01) was reported for a porosity of 90%^53^. For PSi with ~60% porosity, which is a reliable substrate for re-epitaxy, an estimation *s* ~ 0.1 is given. This value of *s* is much smaller than those of the SOI substrate tested in the part A.

**Figure 4:**
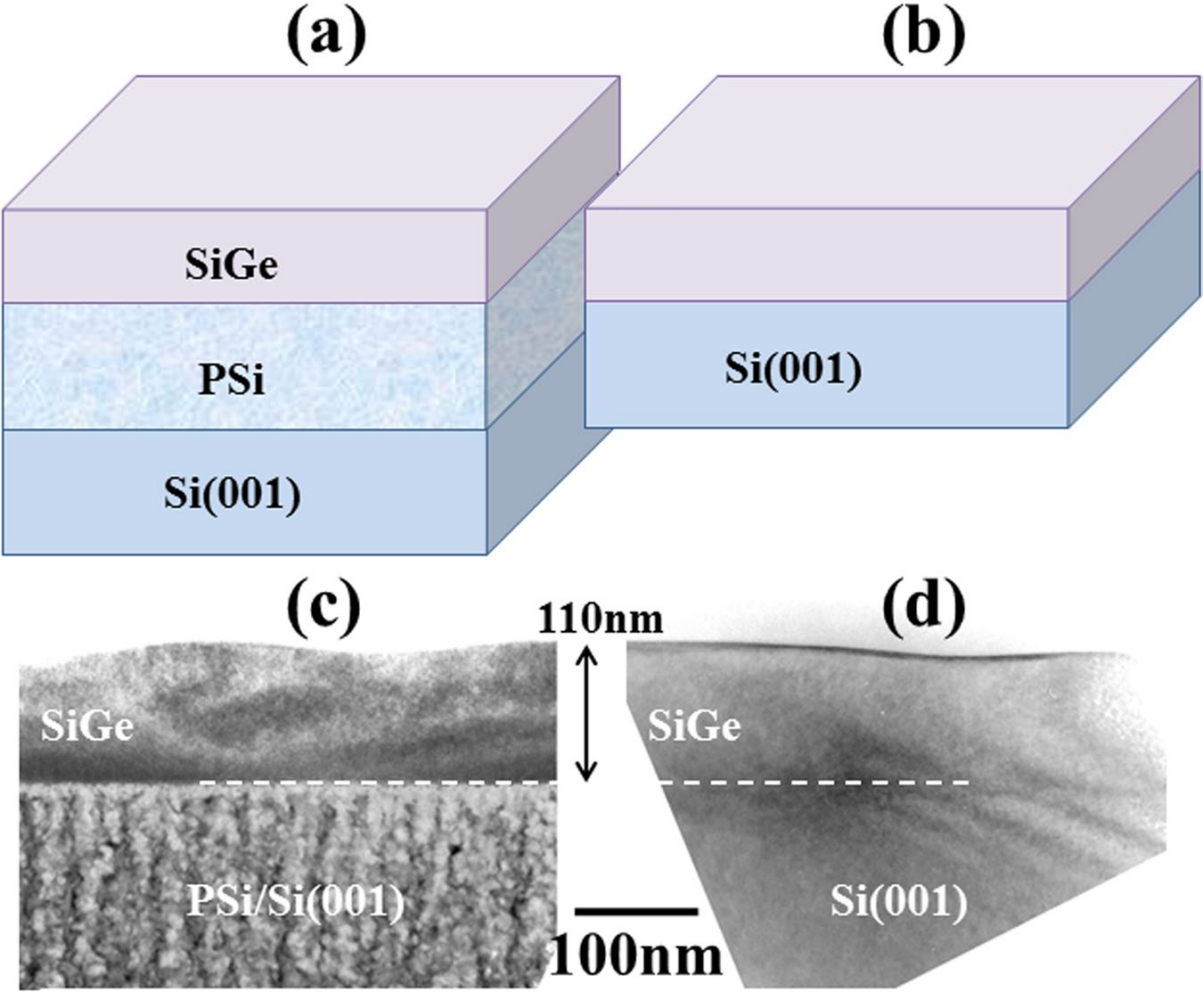
(**a**,**b**) give the schematic representation of the systems Si_1−x_Ge_x_/PSi and Si_1−x_Ge_x_/Si respectively; (**c**,**d**) are the corresponding TEM cross-section images for Si_1−x_Ge_x_ (x = 0.15 − h = 110 nm). The top surface of the epitaxial layers is slightly corrugated in the two situations.

We compared the morphological evolution of SiGe layers (x = 0.15) on Si(001) and on PSi(001) with 60% porosity. After deposition of 110 nm we can see that the surface morphology is about the same on the two substrates with an undulation of small amplitude and large wavelength (A = 10 nm; λ ~ 300 nm) (Fig. 4). The undulation was measured using AFM images not shown here (see^54^ for more details on the measurement process). The values obtained are representative of the commonly observed ATG instability during the growth of SiGe on Si(001)^55^.

We can conclude that in these experimental conditions, while the PSi substrate is much softer, it has no influence on the development of the instability^41^. If we observe the corresponding simulation (for *s* = 0.1), we see that the instability could be considerably enhanced depending on $(\,\bar{h}/{l}_{0})$.

#### In our experimental conditions

*h*_*SiGe*_ = 110 *nm*, *e*_*Si buffer*_ = 20 *nm*, *x* = 0.15, $\,\bar{h}=130\,\,nm\,\,\,$we then have $\bar{h}/{l}_{0}=1.4$ (Fig. 1b). Even if an increase of the instability is predicted theoretically (*s* = 0.1 corresponds to the black curve, as compared to the red one for bulk silicon *s* = 1), it cannot be quantitatively estimated experimentally even if a slight increase of the instability amplitude is visible on the TEM cross-section image of the SiGe/PSi system as compared to the SiGe/Si (comparison of the undulation amplitude between Fig. 4a and b). The small difference between the two samples is explained by the growth fluctuations that could hide the different evolutions of the instability to a certain extent.

These results demonstrate that in real experimental conditions for the SiGe/CS system, the softness (*s* = 0.1) of the PSi substrate has a very small effect on the development of the instability and is not sufficient to fully suppress the instability. More generally, for SiGe heteroepitaxy, it seems very difficult to get an effect of a compliant substrate unless we could realise the epitaxy on a much harder substrate. Typically values of *s* = 10 and $\bar{h}/{l}_{0}=0.3$ would permit this cancellation (see Fig. 1a). Such values cannot be reached with the SiGe/porous-Si or SiGe/SOI systems.

### Effect of a pre-strained substrate: the case of high temperature porous silicon

We consider now another situation where the substrate is both soft and tensely pre-strained. This situation corresponds to the PSi heated at High Temperature (HTPSi). It was demonstrated previously that during heating (at *T* >950 °*C*) a tensile strain is built-in HTPSi due to the desorption of the volatile species incorporated in PSi during the electrochemical fabrication process^56^. The pre-strain produces a decrease of the effective misfit experienced by the deposited SiGe layer. In this situation, the pre-strain has a dual role first the inhibition of the development of the ATG instability and second suppression of the nucleation of dislocations^45^.

**Figure 5:**
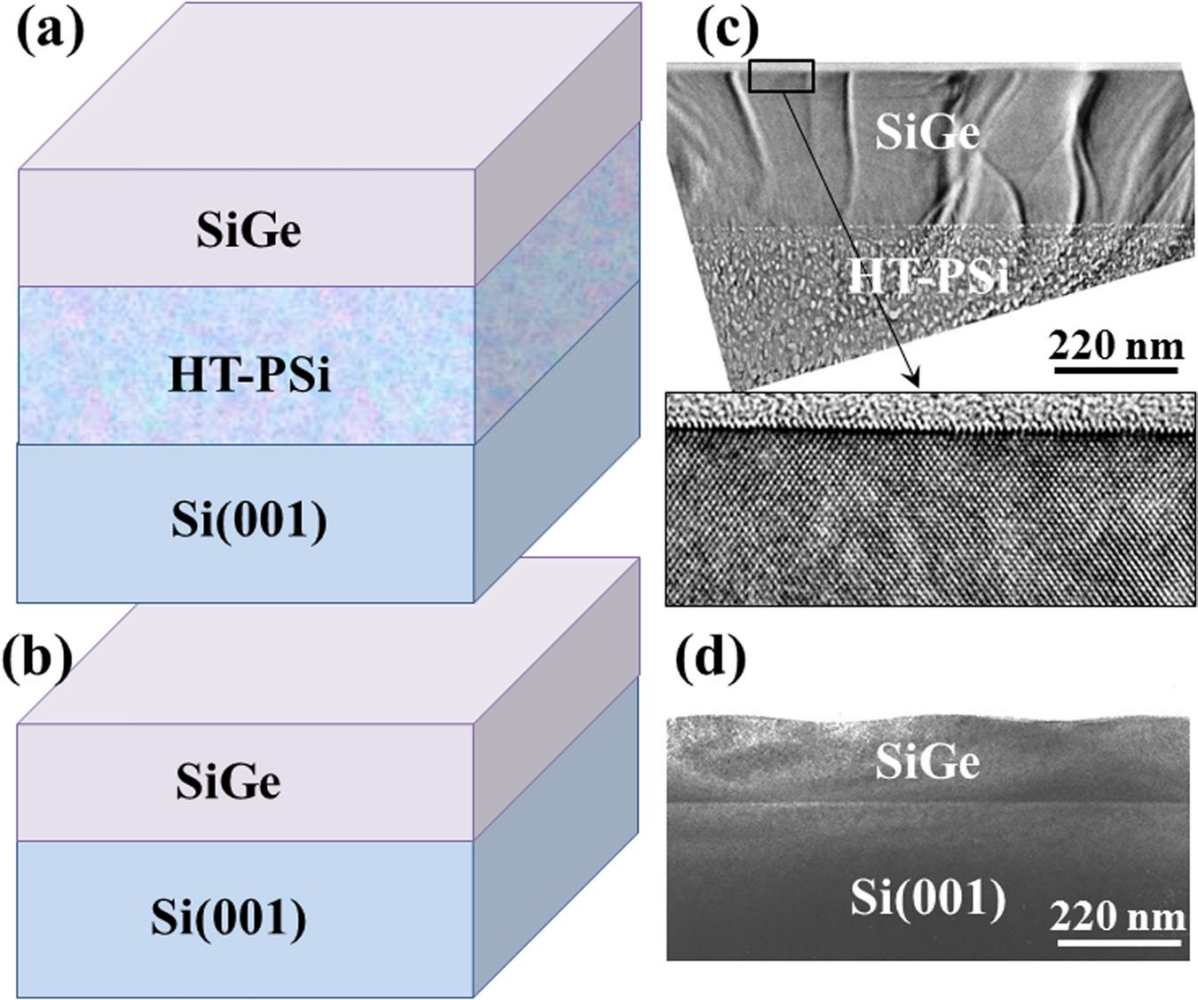
Schematic representations of the epitaxial systems of: (**a**) Si_1−x_Ge_x_/HTPSi/Si(001) and (**b**) Si_1−x_Ge_x_/Si(001); Corresponding TEM cross-section images of SiGe_x_ epitaxial layers (x = 0.15) on (**a**) HTPSi where the surface is totally flat after the deposition of h = 250 nm; (**b**) Si(001), where the surface is already corrugated after the deposition of h = 150 nm

**Figure 6:**
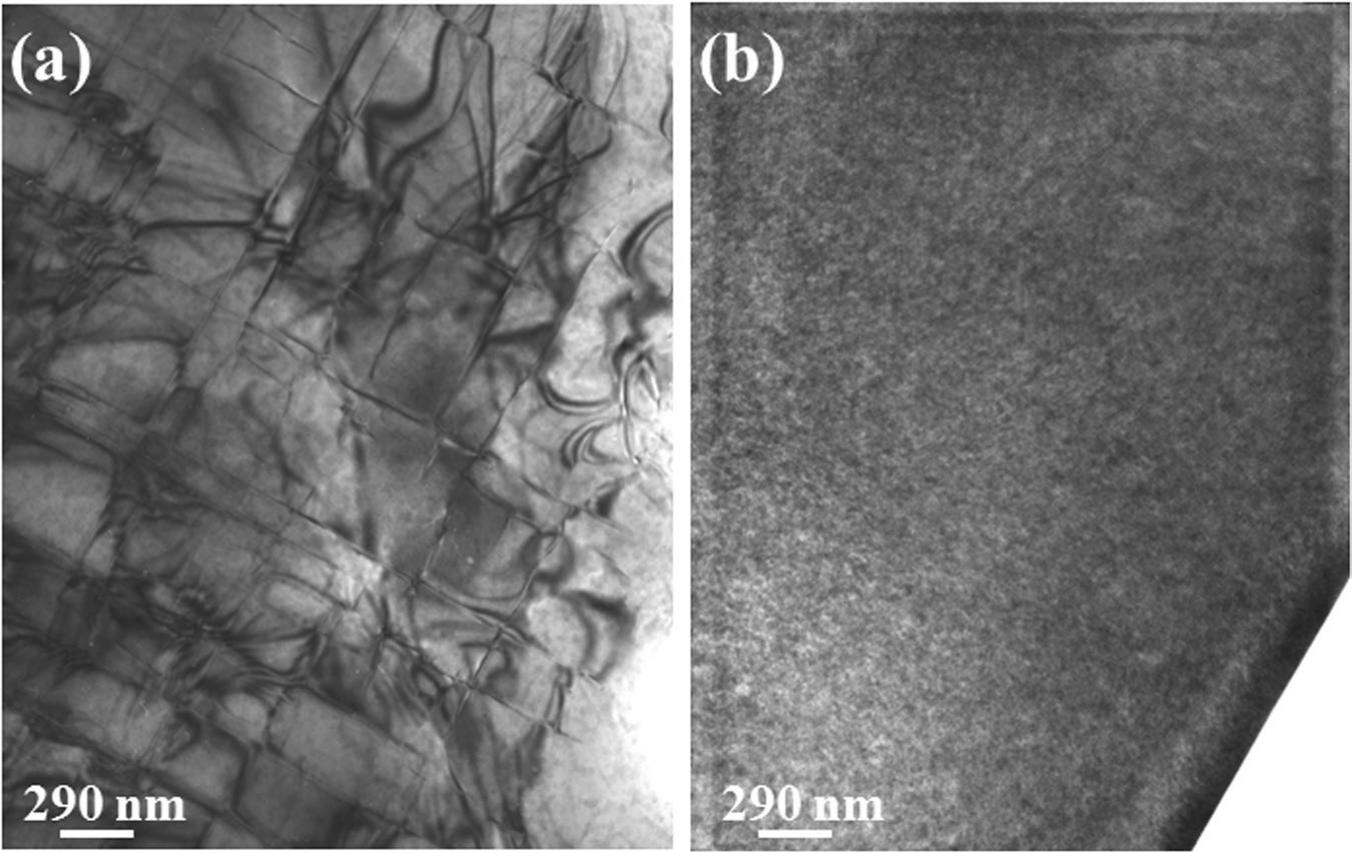
TEM plane view observations of: (**a**) SiGe_x_/Si(001); (**b**) SiGe_x_/HT-PSi/Si(001) heterostructures for x = 0, 15 and a deposited thickness of 250 nm.

For example, a comparison of Si_1−x_Ge_x_ layers (x = 0.15) deposited on HTPSi and on nominal Si(001) is given in Fig. 5. On HTPSi, the SiGe layer remains fully flat and free of extended defects up to h = 250 nm (Fig. 5c) while on Si(001) it is already corrugated for h = 150 nm (Fig. 5d). When h = 250 nm, the epitaxial film SiGe/Si(001) exhibit misfit dislocations that appear as square arrays of lines on plane view TEM image (Fig. 6a) while no dislocation are observed on SiGe/HT-PSi/Si(001) heterostructure (Fig. 6b). Similar results were already observed in a previous study^50^. In these experimental conditions, the results are assigned only to the prestrain while the softness of HTPSi has been shown to have no effect (as already discussed in the previous parts).

We deduce from this section that a small tensile prestrain in the substrate could efficiently suppress the instability and has a significant visible effect. Moreover, we also observed that the tensile pre-strain inhibits the nucleation of dislocations. The effects observed agree with the theoretical calculations without considering any role of the softness. This result again confirms the negligible effect of the softness on the development of the instability at the opposite of a small prestrain.

## Conclusions

In conclusion, we have shown that the effect of a compliant substrate on the morphological evolution of epitaxial layers is quite complex and could either amplify (when the substrate is soft) or inhibit (when the substrate is rigid) the ATG growth instability. However, this effect may be fully suppressed by the normalized thickness of the system $\bar{h}/{l}_{0}$. It is then crucial to fully control these parameters and to understand their respective effects in specific growth conditions for controlling strain engineering,relaxation and morphological evolution. The fundamental concepts of the model have been shown to be valid for the epitaxial growth of SiGe on silicon on insulator (SOI), on as grown porous silicon (PSi) and on high temperature annealed porous silicon (HTPSi). In all the situations a good agreement between the experimental results and the theoretical predictions are obtained.

More precisely, we have shown that there is almost no effect of the softness on the development of the SiGe ATG growth instability in normal experimental conditions due to the inhibiting effect of $\bar{h}/{l}_{0}$. This result has been evidenced for two different soft substrates, SOI (*s* = 0.5) and porous silicon (*s* = 0.1). We also demonstrated that while a soft substrate is quite ineffective, at the opposite a small tensile pre-strain (for instance on HTPSi) fully inhibits both the development of the ATG instability and the nucleation of dislocations. All the concepts developed here could be applied to many other systems and could serve to facilitate heterogeneous epitaxy on silicon.

## References

[1] Paskiewicz DM, Tanto B, Savage DE, Lagally MG (2011). Defect-Free Single-Crystal SiGe: A New Material from Nanomembrane Strain Engineering. Acs Nano.

[2] Khang DY, Jiang HQ, Huang Y, Rogers JA (2006). A Stretchable Form of Single-Crystal Silicon for High-Performance Electronics on Rubber Substrates. Science.

[3] Song Y (2011). Mobility Enhancement Technology for Scaling of CMOS Devices: Overview and Status. J. Electronic Mat..

[4] Saad, I., *et al*. Impact of strain and DP position on the performance of Vertical Strained-SiGe Impact Ionization MOSFET incorporating dielectric pocket (VESIMOS-DP). Published in: *TENCON 2013 - 2013 IEEE Region 10 Conference* **1–3**, 1876–1882 (2014)

[5] Vedula RP (2015). A. Optimal Ge/SiGe nanofin geometries for hole mobility enhancement: Technology limit from atomic simulations. J. Appl. Phys..

[6] Boztug C, Sanchez-Perez JR, Cavallo F, Lagally MG, Paiella R (2014). Strained-Germanium Nanostructures for Infrared Photonics. Acs Nano.

[7] Peidous IV, Lottes C, Jost C (2014). SOI and Bulk FinFET Alternatives from the Perspective of Strain Engineering. ECS Transactions.

[8] Carron V (2013). Y. Source and Drain Contact Module for FDSOI MOSFETs: Silicidation and Strain Engineering. ECS Transactions.

[9] Ma XB (2010). Strain Stability and Carrier Mobility Enhancement in Strained Si on Relaxed SiGe-on-Insulator. J. Electrochem. Soc..

[10] Wang GL (2013). Optimization of SiGe selective epitaxy for source/drain engineering in 22 nm node complementary metal-oxide semiconductor (CMOS). J. Appl. Phys..

[11] Lee CF, He RY, Chen KT, Cheng SY, Chang ST (2015). Strain engineering for electron mobility enhancement of strained Ge NMOSFET with SiGe alloy source/drain stressors. Microelec. Engineering.

[12] Engsiew K, Hamid A, Khairiah F, Razali I (2014). Future of Nanoscale Strained Si/Si x Ge1–x Metal-Oxide Semiconductor Field-Effect Transistor for Performance Metric Evaluation: A Review. J. Nanoelectronics Optolectronics.

[13] Falub CV (2012). Scaling Hetero-Epitaxy from Layers to Three-Dimensional Crystals. Science.

[14] Naffouti M (2016). Fabrication of poly-crystalline Si-based Mie resonators via amorphous Si on SiO2 dewetting. Nanoscale.

[15] Aqua JN, Berbezier I, Favre L, Frisch T, Ronda A (2013). Growth and self-organization of SiGe nanostructures. Phys. Rep..

[16] Zaumseil P (2012). Compliant Si nanostructures on SOI for Ge nanoheteroepitaxy—A case study for lattice mismatched semiconductor integration on Si(001). J. Appl. Phys..

[17] Yin H (2005). Tunable uniaxial vs biaxial in-plane strain using compliant substrates. Appl. Phys. Lett..

[18] Ayers JE (2008). Compliant substrates for heteroepitaxial semiconductor devices: theory, experiment, and current directions. J. Electronic Mat..

[19] Powell AR, Iyer SS, LeGoues FK (1994). New approach to the growth of low dislocation relaxed SiGe material. Appl. Phys. Lett..

[20] Brunner K, Dobler H, Abstreiter G, Schäfer H, Lustig B (1998). Molecular beam epitaxy growth and thermal stability of Si1−xGex layers on extremely thin silicon-on-insulator substrates. Thin Solid Films.

[21] Zaumseil P, Yamamoto Y, Schubert MA, Schroeder T, Tillack B (2014). Heteroepitaxial growth of Ge on compliant strained nano-structured Si lines and dots on (001) silicon on insulator substrate. Thin Solid Films.

[22] Sookchoo P (2013). Strain engineered SiGe multiple-quantum-well nanomembranes for far-infrared intersubband device applications. Acs Nano.

[23] Bonera E, Gatti R, Isella G, Norga G, Picco A (2013). Dislocation distribution across ultrathin silicon-on-insulator with epitaxial SiGe stressor. Appl. Phys. Lett..

[24] Ye L (2015). Ge-on-insulator wafer with ultralow defect density fabricated by direct condensation of SiGe-on-insulator structure. Appl. Surf. Sci..

[25] Liu X (2010). Modified postannealing of the Ge condensation process for better-strained Si material and devices. J. Vac. Sci. & Technol. B.

[26] Ren S (2012). A Ge/SiGe quantum well waveguide modulator monolithically integrated with SOI waveguides. IEEE Photon. Technol. Lett..

[27] Michel J, Liu J, Kimerling LC (2010). High-performance Ge-on-Si photodetectors. Nature Photonics.

[28] Liang D, Bowers JE (2010). Recent progress in lasers on silicon. Nature Photonics.

[29] Park I-S (2014). Dielectric function of Si1−xGex films grown on silicon-on-insulator substrates. J. Appl. Phys..

[30] Torres Sevilla GA (2014). Flexible Nanoscale High-Performance FinFETs. Acs nano.

[31] Moriya R (2014). Cubic Rashba Spin-Orbit Interaction of a Two-Dimensional Hole Gas in a Strained- Ge/SiGe Quantum Well. Phys. Rev. Lett..

[32] Zhang L, Agarwal AM, Kimerling LC, Michel J (2014). Nonlinear Group IV photonics based on silicon and germanium: from near-infrared to mid-infrared. Nanophotonics.

[33] Suh J, Nakane R, Taoka N, Takenaka M, Takagi S (2011). Highly strained-SiGe-on-insulator p-channel metal-oxide-semiconductor field-effective transistors fabricated by applying Ge condensation technique to strained-Si-on-insulator substrates. Appl. Phys. Lett..

[34] Cassé M (2012). Experimental Investigation of Hole Transport in Strained Si1−xGex/SOI pMOSFETs-Part I: Scattering Mechanisms in Long-Channel Devices. *IEEE Trans*. On Electron Devices.

[35] Lin G-Y (2016). Strain evolution of SiGe-on-insulator fabricated by germanium condensation method with over-oxidation. Mat. Scie. in Semicond. Proc..

[36] Souriau L, Terzieva V, Vandervorst W, Clemente F, Brijs B (2008). High Ge content SGOI substrates obtained by the Ge condensation technique: A template for growth of strained epitaxial Ge. Thin Solid Films.

[37] Smith, C. E. *et al*. Dual channel FinFETs as a single high-k/metal gate solution beyond 22 nm node. *in IEDM Tech. Dig*., 309–312 (2009)

[38] Jiang Y (2008). Reduced carrier backscattering in heterojunction SiGe nanowire channels. Appl. Phys. Lett..

[39] Tezuka T, Nakaharai S, Moriyama Y, Sugiyama N, Takagi N (2005). High-mobility strained SiGe-on-insulator pMOSFETs with Ge-rich surface channels fabricated by local condensation technique. IEEE Elec. Device Lett..

[40] Magoariec H, Danescu A (2009). Modeling macroscopic elasticity of porous silicon. Physica Statas Solidi C, Current topics in Solid State Phys..

[41] Calabrese G (2014). Ge growth on porous silicon: The effect of buffer porosity on the epilayer crystalline quality. Appl. Phys. Lett..

[42] Al-Douri Y, Ahmed NM, Bouarissa N, Bouhemadou A (2011). Investigated optical and elastic properties of Porous silicon: Theoretical study. Materials and Design.

[43] Bisi O, Ossicini S, Pavesi L (2000). Porous silicon: a quantum sponge structure for silicon based optoelectronics. Surf. Sci. Rep..

[44] Kang ZH, Liu Y, Lee ST (2011). Small-sized silicon nanoparticles: new nanolights and nanocatalysts. Nanoscale.

[45] Halimaoui A (1991). Electroluminescence in the visible range during anodic oxidation of porous silicon films. Appl. Phys. Lett..

[46] Buttard D (1999). Porous silicon strain during *in situ* ultrahigh vacuum thermal annealing. J. of Appl. Phys..

[47] Berbezier I, Halimaoui A (1993). A microstructural study of porous silicon. J. of Appl. Phys..

[48] Aqua JN, Favre L, Ronda A, Benkouider A, Berbezier I (2015). Configurable Compliant Substrates for SiGe Nanomembrane Fabrication. Cryst. Growth Design.

[49] Novikov PL, Bolkhovityanov YB, Pchelyakov OP, Romanov SI, Sokolov LV (2003). Specific behaviour of stress relaxation in GexSi1−x films grown on porous silicon based mesa substrates: computer calculations. Semicond. Sci. Technol..

[50] Blanchard NP, Nazarov A, Colinge JP, Balestra F, Raskin JP, Gamiz F, Lysenko V (2011). Engineering Pseudosubstrates with Porous Silicon Technology. Semiconductor-On-Insulator Materials for Nanoelectronics Applications. Engineering Materials..

[51] Aouassa M (2012). Ultra-thin planar fully relaxed Ge pseudo-substrate on compliant porous silicon template layer. Appl. Phys. Lett..

[52] Spencer BJ, Voorhees PW, Davis SH (1991). Morphological instability in epitaxially strained dislocation-free solid films. Phys. Rev. Lett..

[53] Bellet D, Lamagnère P, Vincent A, Bréchet Y (1996). Nanoindentation investigation of the Young’s modulus of porous silicon. J. Appl. Phys..

[54] Berbezier I, Ronda A, Portavoce A (2002). SiGe nanostructures: new insights into growth processes. J. of Phys.: Cond. Matter.

[55] Berbezier I, Ronda A, Volpi F, Portavoce A (2003). Morphological evolution of SiGe layers. Surf. Science.

[56] Berbezier I (2014). Accommodation of SiGe strain on a universally compliant porous silicon substrate. Phys. Rev. B.

